# Academic generational differences in Chinese universities: Work preferences, research behaviors and research outputs

**DOI:** 10.1371/journal.pone.0339668

**Published:** 2025-12-31

**Authors:** Yangyang Cui, Hui Guo

**Affiliations:** School of Education, Huazhong University of Science and Technology, Wuhan, Hubei, China; Instituto Tecnologico Autonomo de Mexico, MEXICO

## Abstract

Utilizing data from the 2018 Survey of Academic Profession in the Knowledge Society in China, this study examines generational divergences in work preferences and research behaviors. It further analyzes how these factors relate to the research outputs. The findings indicate that the younger generation serves as the predominant force in research production, exhibiting pronounced research preferences, dedicating more time to research activities, collaborating less, and producing the highest volume of high-impact publications (HIPs). Conversely, the older generation acts as the ‘social service representative,’ devoting more time to social service, collaborating more extensively on research, and balancing teaching and research responsibilities. The middle generation demonstrates research behaviors exhibiting intermediate associations between the older and younger cohorts. International cooperation correlates with elevated HIPs across all generations, showing the strongest association in the older generation. Research time investment positively correlates with HIPs among the younger and middle generations, while research-oriented preferences exhibit associations with HIPs among the younger and older generations. Consequently, institutions should recalibrate evaluation systems by integrating senior academics’ expertise and fostering intergenerational networks that recognize diverse contributions.

## Introduction

Since China’s reform and opening up policy, transformative higher education reforms have prioritized enrollment expansion, the development of world-class universities through initiatives like the 985 Project, and enhanced academic competitive mechanisms. The 1999 enrollment expansion policy accelerated quantitative growth, while the contemporaneous 985 Project targeted institutional excellence. The 985 Project, launched in 1999 alongside the expansion policy, specifically promoted research infrastructure development. Within this context, high-impact international publications (HIPs) became a dominant performance metric in China’s academia, mirroring global academic capitalism trends where intensified competition and managerial governance restructured faculty incentives [1,2].

Building on earlier reforms, China’s post-2014 world-class university initiatives further entrenched these global trends through structural reforms. State-led mechanisms institutionalized globally benchmarked research excellence frameworks, triggering the nationwide restructuring of faculty evaluation systems through performance-linked compensation. These reforms systematically embedded an audit culture into academic labor, intensifying metric-driven governance [3]. This prioritized HIPs production, formalizing metrics like CNS journals (Cell, Nature, Science), Nature and Science subjournals, and JCR Q1 publications in promotion criteria [4,5]. Underpinned by New Public Managerialism principles—particularly accountability infrastructures—China’s higher education ecosystem underwent a fundamental realignment, reconstructing faculty research priorities and behavioral incentives.

Within this audit-driven transformation, China’s faculty demographics shifted significantly. The government’s 2010 launch of the Young Thousand Talents Program (YTTP) recruited over 2,000 exceptional young scholars by 2017, significantly diversifying faculty composition [6]. Meanwhile, return rates among international graduate surged from 29.5% (2005) to 79.1% (2017), demonstrating rapid talent repatriation. Returnees currently form a sizable portion of early-career academics [7], and their familiarity with international publication standards has been instrumental in normalizing the prioritization of JCR Q1 outputs.

Faculty cohorts exhibit divergent academic socialization trajectories rooted in distinct generational contexts (e.g., pre-/post-massification eras) and educational provenance (domestic vs. returnee). These trajectories generate cohort-specific professional schemas that differentially mediate responses to audit-driven governance. Within China’s metric-centered regime, institutions are increasingly prioritizing research output. This emphasis is reflected in personnel appointments, promotion evaluations, and salary incentives aimed to enhance research productivity. Consequently, generational differences significantly predict variations in research productivity through divergent adaptations to performance incentives.

Academic generational differences manifest globally, with extensive international scholarship documenting their varied expressions In contrast, Chinese academia has paid comparatively less attention to this phenomenon. However, rapid sociocultural transformations and higher education reforms in China may amplify intergenerational distinctions among faculty. A nuanced understanding of these differences is essential for designing effective faculty management frameworks. Leveraging 2018 data from the Academic Profession in Knowledge Society (APIKS) survey, this study addresses three research questions: 1*. How do work preferences and research behaviors differ across academic generations (younger, middle, older) among Chinese university faculty? 2. How do these differences in work preferences and research behaviors correlate with research productivity, particularly international publication output? 3. How do academic generations moderate the relationships between work preferences, time investment, collaboration, and research productivity?* Our findings aim to provide actionable insights for the evidence-based refinement of faculty management systems.

## The literature review and research hypothesis concerning generational differences

The classification of academic generations commonly employs three criteria: years of professional experience, age, and rank. Scholars in South Korea and Argentina predominantly use years of work experience for generational classification. In Korea, scholars delineate the young, middle, and older generations as those with less than 10 years, 10–20 years, and more than 20 years of experience [8,9]. Argentine scholars apply distinct thresholds: early-career (<20 years), mid-career (20–35 years), and established scholars (>35 years) [10]. The United States, Poland, and Norway utilize age-based demarcations: emerging scholars (<40 years), mid-phase (40–49 years), and senior academics (≥50 years) [11–13].

Comparative studies reveal significant generational differences in educational experiences, work preferences, work time, research collaborations, and research outputs. Among Korean academics, younger generations show significantly higher rates of doctoral degrees and postdoctoral training versus senior faculty. They exhibit stronger research preferences, allocate more time to teaching and administration, and reduce research time, yet they produce higher research outputs, particularly in international journals [9,13,14]. In the US, younger academics allocate most time to research and service, while middle and older generation faculty focus on management, with the older generation prioritizing teaching. The middle generation demonstrates the highest research output [11]. In Argentina, younger faculty show stronger research preferences than older generations but participate less in domestic and international collaborations. Conversely, the older generation prioritizes teaching and dedicates more time to management, instruction, and service, while the middle generation maintains greater teaching-research balance, often alleviating research pressures through international collaborations [10]. In Norway, younger faculty prefer research, invest more time in it, and engage more in international collaborations, whereas the older generation achieves the highest research output [12,15]. In Poland, younger generation similarly favors research, all of three cohorts devote more time to teaching than to research with minimal intergenerational differences. The older generation, however, participates more actively in international cooperation and slightly surpasses others in research output [12,16]. Collectively, these cross-national patterns confirm systematic generational variations, revealing both convergent and divergent adaptation strategies. Overall, younger generation exhibits stronger research orientation, invest more time and effort into research, and achieve higher research outputs. Conversely, the older generation allocates more time to administrative responsibilities while maintaining stronger research collaboration engagement. This study aims to compare generational differences in work preferences, time investments, and research collaborations, examining generation-productivity associations, while controlling for covariates to identify key productivity predictors.

Research productivity correlates with distinct factors among academic generations. Previous studies employ metrics including three-year publication counts, SSCI/SCI-indexed articles, and paper quality index to evaluate generation’s research productivity. In South Korea, domestic doctoral degrees correlate with younger generation productivity, while international doctoral degrees are associated with older generation output. Postdoctoral experience, male, and senior academic titles show specific correlations with younger generation productivity. Performance-oriented institutional cultures correlate with productivity in both middle and older generations [9]. Research preference correlates with productivity across all generations, showing the strongest effects among younger generation [8,17]. International collaboration strengthens productivity correlations for Norway’s younger generations [18]. For Chinese academics, research cooperation—measured by collaboration rate/partner numbers —shows strong productivity correlations for younger generations in educational disciplines. This aligns with older generations’ established resources and lower promotion pressure, which coincide with reduced innovation motivation and weaker cooperation-output correlations [19]. Teaching-research time investments consistently correlate with among Chinese academics across ranks, with research time positively associating and teaching time negatively associating with output [20].

Existing literature indicates variability in the association strength between educational experiences/institutional policies and research output across academic generations. In contrast, research collaboration and time investment show more consistent associations with productivity across generations. Consequently, this study examines personal agency factors—work preferences, research collaboration, and research time—across academic generations in relation to productivity. Hypotheses specifying directional associations are: Among younger, middle, and older generations, research preference, research time investment, and research collaboration (international**/**domestic**/**intramural) will be positively associated with HIPs quantity. Conversely, teaching time allocation will show negative associations with HIPs.

## Methods

### Variables and analyzing strategy

#### Dependent variable.

Research productivity. Given the transformations in China’s higher education system and contemporary context, this study focuses on “HIPs.” This was quantified by the “number of papers published in SCI/SSCI journals over the past three years,” [21] and measured using a count variable.

#### Independent variables.

Academic generations. Academic generations constituted a categorical variable based on age, with birth year serving as the classification metric (age calculated as 2018 minus birth year) [22]. Following prior methodology [11,12], participants comprised three generations: Younger Generation (YG), defined as those under 40 years of age and typically within a decade post-doctorate [12]; Middle Generation (MG), comprising individuals aged 40–49 years; and Older Generation (OG), defined as individuals aged 50 years or older. For regression analyses, this generational variable was coded as follows: 1 = YG (reference), 2 = MG, 3 = OG.

Work preference. Work preference was classified as a categorical variable, assessed using a single item on a 4-point Likert item: 1 = ‘Mainly teaching’ (reference); 2 = ‘Teaching and research, but more inclined to teaching’; 3 = ‘Teaching and research, but more inclined to research’; 4 = ‘Mainly research’.

Work time investment. All work time investment variables—including weekly research time, teaching time, social service time, academic management time, and total work time—were recorded as continuous variables in hours per week.

International collaboration. International collaboration was operationalized as a binary variable based on the question, ‘Who were your partners for this academic year?’ Response options included international peers, external domestic collaborators, and on-campus faculty (multiple selections allowed). This variable was coded as 1 for ‘Yes’ (indicating international collaboration) and 0 for ‘No’ (indicating no international collaboration).

#### Control variables.

To account for potential confounding factors that might also influence publication output, several control variables were included in the model: gender, institution level, discipline, academic rank, and doctoral degree completion status. Institution level was controlled given significant variations in resource allocation, incentive structures, and research cultures. Discipline was controlled for field-specific variations in collaboration patterns and research opportunities [23,24]. Academic rank was controlled to address career-stage evolution in collaboration networks and research capacity. Gender and doctoral training background correlated with differential research capacity and collaboration network structures. Full operational definitions and measurement scales are detailed in Table 1.

**Table 1:** Description of variables.

Independent variable	Type of variable	Measurement	Variable value
Academic generation	Categorical/dummy	Year of birth	1 = Individuals under 40 years years of age were categorized as YG 2 = those aged 40–49 as MG, 3 = individuals aged 50 and above as OG
Work preference	Categorical/dummy	Primary interest in “teaching and research”	1 = Mainly teaching; 2 = Teaching and research, but more inclined to teaching; 3 = Teaching and research, but more inclined to research; 4 = Mainly scientific research
Work time investment	Continuous	The number of hours spent in teaching activities in an average week	Integer between 0 and 126, continuous variable
		The average number of hours a week spent on scientific research	Integer between 0 and 126, continuous variable
		The number of hours spent on social service activities in an average week	Integer between 0 and 126, continuous variable
		The number of hours spent in academic administration activities in an average week	Integer between 0 and 126, continuous variable
Research collaboration	Binary/dummy	Target of cooperation (on-campus cooperation, domestic cooperation, international cooperation)	0 = no, 1 = Yes,
**Dependent variables**			
High-impact international publications	Count	Number of papers published in SCI/SSCI journals over the past three years	Integer
Gender	Binary/dummy	Gender	1 = Female, 2 = male
Rank	Categorical/dummy	rank	1 = Professor, 2 = associate professor, 3 = lecturer
Institutional level	Categorical/dummy	Institutional level	1 = “985 Project”universities, 2 = “211 Project”universities, 3 = Ordinary college
Discipline	Categorical	The current work belongs to the discipline	1 = Humanities, 2 = social sciences, 3 = natural science, 4 = engineering, 5 = others
Phd	Categorical	The country or region where the doctoral degree is located	1 = Domestic doctor, 2 = overseas doctor, 3 = none

The count-based outcome variable –HIPs– exhibited non-negative integer values with significant overdispersion (variance [8.2]> mean [4.0]), violating Poisson assumptions (variance = mean). We therefore employed negative binomial regression (NBRM), with robust standard errors (Stata’s vce(robust)), to address the overdispersion and heteroscedasticity. This model introduces a dispersion parameter (α) to explicitly accommodate extra-Poisson variation. The general form of the negative binomial regression model is expressed as:


$$\text{log}\left(\text{E}\left[Y_{i}\right]\right)=\beta_{\text{0}}+\beta_{\text{1}}\left({AG}_{i}\right)+\beta_{\text{2}}\left({RT}_{i}\right)+\beta_{\text{3}}\left({WP}_{i}\right)+\beta_{\text{4}}\left({IC}_{i}\right)+\sum \beta_{k}({Control}_{ki})$$

where ${\text{Y}}_{\text{i}}$ represents the publication count for scholar I, $\text{E}[{\text{Y}}_{\text{i}}]$ is the expected count, and *β* coefficients represent the change in the log of the expected count associated with a one-unit increase in the predictor, holding other variables constant. The exponentiated coefficients (${\text{e}}^{\beta }$) can be interpreted as incidence rate ratios (IRRs).

Control variables were incorporated in all regression models. Analyses proceeded in three phases: Phase 1 employed descriptive statistics and tests of differences to characterize academic generations across work preferences, work time, and research collaboration. Phase 2 implemented negative binomial regression on the full sample [25], modeling HIPs as the dependent variable with relevant controls. This quantified the incidence rate ratios (IRRs) and their significance regarding the association between academic generations on HIPs. Phase 3 stratified the sample into three predefined academic generations. Identical negative binomial models were executed separately for each cohort, maintaining consistent outcome and control variables to assess cohort-specific IRR magnitudes and significance levels. Analyses utilized SPSS 20.0 and STATA 14.

### Data source

Data were derived from the 2018 Academic Profession in Knowledge Societies Survey (APIKS) conducted in mainland China. Administered from 1 June to 31 August 2018, the APIKS survey distributed 6,070 questionnaires online, yielding 2,632 valid responses. It encompassed 120 public undergraduate institutions covered 22 provinces, stratified into 40 research universities and 80 four-year general undergraduate colleges. Instruments captured faculty demographics, educational trajectories, teaching loads, research productivity, administrative duties, and external engagement, encompassing all study variables. After listwise deletion of cases with missing research output data, the analytical sample included 2,305 valid cases. To enhance representativeness, we weighted the data using auxiliary information from known survey totals, (*The precise procedure involves obtaining data from the China Education Statistical Yearbook regarding the number of teachers in public general undergraduate colleges and universities in 2017 across all position levels and age groups, creating a cross term between “position” and “age,” and weighting the sample data by age. The Yearbook of Educational Statistics states that in 2017, the percentages of college instructors under the ages of 39, 49, and 50 were 53.63%, 27.17%, and 19.2%, while the percentages of professors, associate professors, lecturers, and the following positions in public undergraduate colleges and universities across the country were 12.8%, 30%, and 57.2%*), thereby minimizing discrepancies between sample and population structures [26]. Sample details are presented in Table 2. Voluntary participation may introduce potential self-selection bias, and key variables based on faculty self-reports may contain measurement errors.

**Table 2:** Sample composition.

Variable	Category	Count	%
Academic generations (pre-weighted)	YG	971	42.1
	MG	887	38.5
	OG	447	19.4
Gender	Male	1646	71.4
	Female	659	28.6
Rank	Professor	835	36.2
	Associate professor	889	38.6
	Lecturer	581	25.2
Phd	Domestic doctor	1830	79.4
	Overseas doctor	152	6.6
	none	323	14.0
Institutional level	“985 Project”universities	388	16.8
	“211 Project”universities	497	21.6
	Ordinary college	1420	61.6
Discipline	Humanities	191	8.3
	Social sciences	626	27.1
	Natural science	525	22.7
	engineering	680	29.5
	others	286	12.4
	Total	2305	100.0
Academic generations (after weighting)	YG	1236	52.3
	MG	685	29.0
	OG	443	18.7
	Total	2364	100.0

### Ethics statement

The Institutional Review Board of the Institute of Educational Science, Huazhong University of Science and Technology approved this study, conducted in accordance with the Declaration of Helsinki. University faculty aged ≥18 years were recruited, excluding minors. All data were de-identified before analysis to ensure participant anonymity. Informed consent was obtained electronically. On the first page of the online questionnaire, participants were given comprehensive information (goals, methods, and data handling). They confirmed voluntary participation by clicking a link to proceed. Participants could withdraw at any time before submission without penalty.

## Results of data analysis

### Differences in work preferences between academic generations

The chi-square test indicated statistically significant differences in work preferences among academic generations (Table 3). Cramer’s V coefficient (which reflects the effect size) analysis showed a moderate association (V = 0.15, *p* < 0.001), based on conventional interpretation thresholds (weak: V < 0.15; moderate: 0.15 ≤ V < 0.30; strong: V ≥ 0.30). Post-hoc analysis indicated: (a) YG’s research preference significantly exceeded both MG’s and OG’s (*p *< 0.05), and (b) MG’s preference was significantly higher than OG’s (*p *< 0.05). YG showed the strongest research preferences, while OG exhibited balanced teaching-research distribution (ratio ≈ 1:1). MG’s preferences were intermediate between YG and OG.

**Table 3:** Presents work preferences differences across academic generations.

Variable	Mainly teaching	Teaching and research, but leaning towards teaching	Teaching and research, but leaning towards research	Mainly research	Chi-square
	%	%	%	%	
YG	4	23.5	59.2	13.3	105.675***
MG	7.4	27.4	58.8	6.3	
OG	6.6	43.2	46.2	4.1	

*p < 0.05, **p < 0.01, ***p < 0.001, Same below.

### Differences in research behaviors between academic generations

#### Differences in work time between academic generations.

Significant differences emerged across academic generations in **research time, social service time, and total work time (**Table 4**).** Effect sizes were quantified using Cohen’s d [27]. For research time, YG devoted significantly more time than OG and MG (*p* < 0.01), with moderate effect sizes versus OG (d = 0.22) and MG (d = 0.19). No significant MG-OG difference was detected (*p *> 0.05). YG demonstrated the longest research time investment. For social service time, YG devoted significantly less time than OG (*p *< 0.01; *d* = 0.2, medium effect), while OG demonstrated the longest engagement duration. No significant differences occurred between MG-OG or YG-MG dyads (*p *> 0.05). In total work time, YG worked significantly longer than OG (*p* < 0.05; *d *= 0.15, small effect), with YG-MG and MG-OG comparisons revealing non-significant differences (*p* > 0.05). YG maintained longer total work hours than other generations.

**Table 4:** Tests of differences in work time between academic generations.

	Teaching time		Research time		Social service time		Academic management time		Total work time	
	Mean	SD	Mean	SD	Mean	SD	Mean	SD	Mean	SD
1	17.80	16.51	35.10	23.46	4.99	8.75	7.86	12.24	65.75	34.55
2	17.30	15.71	30.97	20.87	5.76	8.13	8.33	13.92	62.37	34.69
3	16.64	15.91	29.44	27.27	6.65	8.21	7.73	11.15	60.45	37.09
F	0.817		11.938***		6.620**		0.412		4.498*	

1 = YG, 2 = MG, 3 = OG.

#### Variations in research collaboration across academic generations.

Chi-square analysis revealed significant differences in **on-campus, international, and domestic collaboration** (*p* < 0.001; Table 5). Despite statistical significance, Cramer’s V indicated weak associations (on-campus: V = 0.13; international: V = 0.11; domestic: V = 0.07; all *p* < 0.001). Post-hoc tests showed: (a) OG > MG > YG for on-campus collaboration (all *p* < 0.05), with OG showing the highest engagement; (b) for domestic collaboration, OG significantly outperformed YG (*p* < 0.05), with no MG-OG or YG-MG difference (both, *p* > 0.05); and (c) for international collaboration, OG exceeded both YG and MG (both, *p* < 0.05), with no YG-MG difference (*p* > 0.05). These results demonstrate OG’s consistently dominant collaboration across all types.

**Table 5:** Tests of differences in research collaboration between academic generations.

Variable	On-campus cooperation		International collaboration		Domestic collaboration	
	No(%)	Yes(%)	No(%)	Yes(%)	No(%)	Yes(%)
YG	38.7	61.3	52.1	47.9	82.4	17.6
MG	30.2	69.8	46.6	53.4	81.9	18.1
OG	22.8	77.2	44.1	55.9	71.5	28.5
χ²	41.311***		10.600**		25.990***	

### The association between academic generations and research output, and differences in relevant factors across cohorts

#### The relationship between academic generations and HIPs.

Model 1 presented in Table 6 displayed incidence rate ratios (IRRs) for generational differences in HIPs. After controlling for institutional characteristics, faculty attributes, and research practices, MG and OG showed lower expected publication counts than YG (MG: IRR = 0.636, 36.4% lower; OG: IRR = 0.522, 47.8% lower). Among the three generational cohorts in Chinese higher education institutions, YG exhibited the highest international publications.

**Table 6:** Regression analysis.

Variable	Model1	Model2	Model3	Model4	Model5	Model6	Model7
MG (refer to the YG)	0.636***	0.634*	0.531**	0.682**			
OG	0.522***	0.464*	0.583*	0.49**			
Men (refer to women)	1.094	1.422*	0.921	1.087	1.102	0.997	1.242
211 Project universities (refer to “985 Project”universities)	0.723**				0.746*	0.679*	0.814
Ordinary college	0.610**				0.635**	0.643**	0.745
Associate Professor (refer to Professor)	0.360***	0.289***	0.449***	0.352***	0.472***	0.413***	0.318***
Lecturer	0.219***	0.154***	0.229***	0.219***	0.312***	0.211***	2.12 × 10 ⁻ ⁸***
Social sciences (refer to Humanities)	2.812**	2.343	1.723	3.732**	2.600*	2.413	4.590*
Science	19.799***	9.88***	15.825***	30.446***	15.825***	27.180***	36.966***
Engineering	13.889***	9.53***	14.234***	20.267***	14.234***	13.817***	14.610***
Others	12.736***	12.736***	11.400***	16.241***	11.400***	14.909***	28.014***
Overseas doctorate (cf. domestic doctorate)	1.476**	1.614**	1.43	1.252	1.505*	1.920*	0.775
without a doctorate	0.299***	0.269*	0.335**	0.315***	0.28***	0.430***	0.346***
Teaching and research, but with a preference for teaching (cf. primarily teaching)	1.399	0.507	2.473	1.424	1.912	0.731	6.713***
Teaching and research, but more research oriented	2.199***	1.07	2.891*	2.345**	2.606*	1.176	13.594***
mainly research	2.588***	1.407	2.58	2.616**	2.387*	1.34	33.041***
Teaching time	0.994	0.988*	0.994	0.993	0.994	0.993	0.994
Research time	1.007***	1.004	1.009**	1.008**	1.005*	1.009*	1.007
On-campus cooperation (cf. no)	0.943	0.651*	0.928	1.031	1.059	0.799	0.736
Domestic cooperation	0.942	1.164	0.922	0.892	1.016	1.174	0.847
International cooperation	1.565***	1.41	1.57**	1.633***	1.313*	1.768***	2.732***
Constant	0.414	1.374	0.36	0.158	0.267*	0.415	0.018***
Alpha	1.007	0.949	0.893	0.988	0.644	1.11	1.48
Observations	2305	388	497	1420	971	887	447

Table 6 presented Models 2–4 analyzing generational associations with expected counts of HIPs across university tiers. YG consistently showed significantly higher expected counts than both MG and OG across all tiers. This institutional-tier stratified analysis served as a robustness check, confirming the findings from the full sample.

#### Associations involving HIPs and YG.

Models 5–7 analyzed associations between various factors and HIPs across three cohorts: YG, MG, and OG. Model 5 revealed that among YG faculty, research preference, research time, and international collaborations were positively associated with HIPs after adjusting for institutional and individual covariates. Teaching time, on-campus and domestic collaborations showed no significant associations. YG faculty preferring ‘research-inclined’ roles demonstrated 2.6-fold higher expected publication counts than teaching-focused peers, while those with ‘primarily research’ preference showed 2.4-fold higher publication rate. Each additional research hour corresponded to 0.5% higher expected publication counts, while international collaborators showed 1.3-fold greater publication counts.

#### Associations involving HIPs and MG.

Model 6 demonstrated among MG faculty, after controlling for institutional characteristics and individual attributes, international collaboration and research time showed significant positive associations with HIPs. Work preferences, teaching time, on-campus, and domestic collaboration showed no significant associations. Each additional research hour was associated with 0.9% higher expected counts of HIPs. MG faculty engaged in international collaborations showed 1.8 times expected counts compared to non-collaborators.

#### Associations involving HIPs and OG.

Model 7 demonstrated, after accounting for institutional characteristics and individual attributes, research-oriented preference and international collaboration were associated with elevated expected counts of HIPs in OG faculty. Research time, teaching time, on-campus collaborations, and domestic collaborations showed non-significant associations. OG faculty with referring ‘primarily research’ exhibited 33.0-fold higher expected publication versus ‘primarily teaching’ counterparts; those with ‘research-inclined’ preference showed 13.6-fold difference in expected publication counts. OG faculty engaged in international collaborations demonstrated 2.7-fold higher expected publication counts than non-collaborators.

#### Variations in influencing factors of HIPs across distinct academic generations.

In summary, international collaboration was significantly positively associaed with HIPs across all generations, strongest in OG and weakest in YG. Research-oriented preferences were positively associated with HIPs in YG and OG, more strongly in OG; but not in MG. Research time showed a marginal positive association with HIPs in YG and MG, but not in OG. Teaching time, on-campus collaboration, and domestic collaboration showed no significant associations with HIPs in any generation.

## Conclusions, discussion, and implications

### Conclusions

Analysis of variances in work preferences, work time, and research collaboration across academic generations, coupled with their associations with research output, revealed distinctive generational scholarly engagement profiles. These cross-sectional estimates demonstrate statistical relationships; they do not support causal inference.

The YG serves as the principal driver of research output, exhibiting pronounced research inclinations, dedicating more time to research activities, collaborating less, and producing the highest volume of prestigious international publications. This is consistent with Kwiek’s [28] findings. In contrast, the OG acts as the ‘social service representatives,’ devoting more time to service activities, collaborating more extensively on research, and balancing teaching and research responsibilities. The MG functions as the transitional cohort, demonstrating moderate research orientation, allocating less research time than the YG while collaborating less than the OG.

Work preferences and research behaviors varied across academic generations and had distinct associations with research productivity. The YG demonstrated stronger research orientation and greater research time investment, both associated with higher HIPs. Conversely, increased research collaboration was significantly associated with higher HIPs among OG. Furthermore, relationships between work preferences, research behaviors, and outputs across generations exhibited a “self-selective” pattern. Specifically, YG faculty with generally low international engagement who collaborated internationally achieved more HIPs than non-participants. Conversely, research-oriented OG scholars produced more HIPs than peers, even with balanced teaching-research preferences. Critically, international collaboration correlated consistently with international publication for all generations, while research orientation showed stronger benefits among YG and OG, especially in the latter. Research time investment demonstrated only modest positive associations with publications among YG and MG.

### Discussion

The reasons and implications behind the above findings are discussed below.

#### Work preferences.

Work preferences’ relationship with HIPs varied significantly across academic generations, primarily co-occurring with institutional pressures [14] and individual autonomy. The younger generation’s research preferences are highly constrained by institutional assessment pressures (e.g., research tournaments for promotion)and limited autonomy. This externally-driven work alignment — focused on meeting quantifiable output metrics —correlated with greater research time allocation [29] but showed a weaker association with substantial HIPs, possibly due to suppressed autonomy. In contrast, OG’s research preferences were associated with early-career socialization and self-selection during professional maturity. Despite correlations between late-career institutional incentives (e.g., performance-based pay) and OG research activities, their autonomy levels remained high. Self-selected preferences (rather than institutional factors) showed the strongest positive association with OG HIPs. MG faculty exhibited covarying promotion needs and constrained autonomy; their moderate research preference showed no significant correlation with increased HIPs. This contrasts with Korean findings [8], where research preference correlated with outputs across all generations. The Chinese context uniquely revealed that preference drivers—external pressure versus internal autonomy—are strongly aligned with high-impact research efficacy.

While YG’s focus on HIPs aligns with contemporary evaluation metrics, excessive indexism jeopardizes intrinsic motivation, compromises research originality and quality, and may neglect young faculty’s vital contributions to collegial talent development [30].

#### International cooperation.

While prior research identified significant correlations between international collaboration and domestic publications among younger academics [20], this study showed a weaker association with HIPs in this cohort. This contrast may reflect contextual differences [18,31]. Unlike discipline-specific prior studies, our broader analysis controlling for characteristics found young researchers engaged internationally correlated with superior overall research performance, aligning with Kyvik and Aksnes [18]. Despite adopting ‘high-yield strategies’ (e.g., cross-border team division) in response to evaluation systems [18], international collaboration in this cohort correlated less strongly with HIPs.

Several factors correlate with this weaker association among young Chinese scholars. **1.** Author-centric incentives: University evaluation systems prioritizing first/corresponding authorship disproportionately disincentivized junior faculty collaboration, as evidenced by survey identifying authorship disputes [32]. 2. Resource constraints: Early career stages with limited specialized equipment and personnel co-varied with diminished capacities for substantive international partnerships. Collaboration patterns frequently aligned with supplementary resource acquisition rather than core intellectual exchange.

#### Research time.

While research time generally correlates with productivity [20,33], our findings reveal generational divergence: increased research time demonstrated significant positive associations with HIPs only among YG and MG faculty. This differential pattern across generations corresponds to distinct research approaches. Among younger and mid-career scholars, extended research hours correlate positively with HIPs. Conversely, among senior faculty, increased research time alone demonstrated non-significant association; whereas their higher international publication links more strongly to strategic academic networking and international collaborations.

### Implications

#### Reforming research ecosystem governance: Evaluation rigor, senior expertise utilization, and cultural agility.

Our study identified a significant positive correlation between research preference and international output, particularly for senior researchers. This aligns with evidence emphasizing the role of intrinsic motivation in academic achievement. We propose the following actionable recommendations:

1. **Revamp Academic Evaluation:** Shift from sole reliance on publication counts to incorporating “research inclination” as a key metric for talent development. Policymakers and institutions should recognize that intrinsic drive fosters sustained high-quality contributions and international engagement. As per the 2021 Central Talent Work Conference, evaluations should prioritize innovation, capability, and contribution. Research-intensive universities should emphasize original innovation and impact, empowering younger scholars with self-directed research. Teaching-intensive institutions should calibrate incentives with their missions, valuing pedagogical impact.
2. **Support Senior Scholars:** Create dedicated platforms to leverage senior researchers’ strong research inclinations. Universities may establish “senior scholar” or “honorary researcher” roles with research space, resources, and project funding. This facilitates knowledge transfer and leverages their expertise for mentorship and innovation.
3. **Cultivate an Exploratory Research Culture:** Develop environments encouraging exploration, accepting experimental approaches, and valuing diverse scholarly outputs (theoretical, applied, pedagogical, societal). Mitigating short-term evaluation pressures could help scholars pursue sustained research across career stages.

#### Generational integration in global science: Targeted networking, contribution valuation, and credit compliance.

Our study revealed a significant positive association between international collaboration and HIPs, with this association being strongest in OG and weakest in YG. This suggests that international collaboration generational differences mediate the relationship between collaboration and publication. Based on this, we propose the following recommendations for China’s policy and practice:

First, to enhance global research engagement, institutions should establish structured international networks through academic platforms and global databases, connecting early-career faculty with global peers. Concurrently, expanding mobility programs (e.g., exchanges, visiting positions) at leading institutions is critical. Repatriated scholars should be incentivized to: (a) build sustained collaboration, (b) present at major conferences, (c) co-develop graduate programs with global partners, and (d) pursue joint grants. Crucially, as international scientific networks exhibit scale-free and small-world properties, strategic positioning of early-career researchers toward pivotal hubs (e.g., elite scholars/institutions) will amplify connectivity [34,35] and significantly increase international publication.

Second, to improve attribution in international collaborations, universities should de-emphasize authorship order and recognize substantive contributions by early-career faculty. Establishing standardized contribution frameworks——such as the Contributor Role Taxonomy (CRediT) adopted by leading journals —correlates with rigorous research credit allocation. This system enhance role transparency and potentially enable equitable recognition of all contributors [36].

#### Sustaining productivity: Research time protection, adaptive workload systems, and teaching-research nexus.

Our regression analysis revealed a statistically significant association between dedicated research time and publication outputs. This highlights the essential role of protected research time for scholarly productivity. To mitigate prevalent concerns regarding excessive teaching and administrative duties, institutions should consider implementing flexible workload allocation systems under national research excellence initiatives such as the ‘Double First-Class’ Initiative. For instance, creating ‘research-intensive tracks’ or ‘research leave’ programs, particularly for early-career researchers, similar to those successfully implemented in leading research universities globally. Critically, these structural reforms must align with broader pedagogical goals: Research evaluations should not only weight contributions more heavily per the Ministry of Education quality mandates, but also assess how research informs teaching innovations, curriculum development, and student mentoring. This dual-focus approach—ensuring adequate research time while integrating inquiry with pedagogy—creates a sustainable ecosystem for academic excellence.

### Limitations

Several study limitations warrant attention in interpreting results. Methodologically, reliance on voluntary participation introduces potential self-selection bias, possibly leading to over-representation of faculty with particular viewpoints (e.g., faculty with heavy workloads may be more inclined to participate). Additionally, self-reported productivity and preference data carry inherent risks of social desirability bias (e.g., overreporting work hours or high-status preferences) and recall inaccuracies. Although data weighting mitigated demographic discrepancies, residual biases may affect absolute estimates of specific variables. However, identified generational difference patterns appear less vulnerable to substantial distortion from these methodological constraints.

A key limitation involves the data’s temporal scope. The 2018 APIKS survey predates transformative events including: (1) the COVID-19 pandemic, and (2) major policy shifts in Chinese higher education (e.g., breaking the Five Onlys (Po Wu Wei) reforms with a new emphasis on diversified evaluation). Extensive evidence indicates the pandemic fundamentally altered academia through: work pattern reorganization (e.g., remote work) [37],research team restructuring [38], and publishing adjustments (e.g., increased preprint adoption [39]). Thus, the documented generational patterns reflect institutional conditions predating 2019, when scientific output metrics and internationalization dominated policy priorities. Critically reflecting on the potential impact, the subsequent policy shifts towards diversified performance assessment may have mitigated the intense pressure on younger scholars to prioritize international publications above other outputs, potentially diminishing the generational gap in publication strategy emphasis observed in 2018. Similarly, pandemic-induced shifts towards virtual collaboration likely altered the barriers and opportunities for international research partnerships, affecting strategies for scholars across generations and possibly equalizing reliance on such collaborations in ways our data could not capture. Therefore, interpretations concerning work motivations, professional preferences, and policy effects require careful contextualization for post-2020 environments.

A further data limitation is its exclusive Chinese focus. Although a cross-national comparative analysis (particularly with countries sharing similar contexts like India) would significantly enhance the generalizability and contextual depth of our findings, such an undertaking was constrained by the lack of granular, methodologically comparable survey data from other national contexts available for this study.

Conceptually, defining generational cohorts mainly by birth year—though practical for cross-study comparison—imposes methodological constraints. This approach cannot capture career-path heterogeneity (e.g., late-stage entrants or transitioned from non-academic sectors). It may also mask variations in professional development trajectories influenced by factors beyond birth year. Future research could benefit from incorporating indicators of a “career age” (e.g., years post-PhD) [40] or career stage alongside chronological age for a more nuanced understanding.

Despite these limitations, this study provides baseline data characterizing faculty in China’s research-intensive universities just prior to major disruptions (pre-2020). This pre-disruption baseline enables systematic assessment of post-2020 changes. We recommend that future studies (i) adopt post-2020 longitudinal data to assess pandemic impacts, (ii) model career-stage trajectory over time, and (iii) corroborate self-reported measures with institutional records when possible; and (iv) prioritize collaborative international surveys to replicate this methodology across diverse contexts (e.g., India, Brazil), enabling systematic comparative analyses of how national policy landscapes and academic cultures shape generational experiences.

## Supporting information

S1 File Dataset: Academic generational differences in chinese universities. (XLSX)
